# Delayed application of silver nanoparticles reveals the role of early inflammation in burn wound healing

**DOI:** 10.1038/s41598-020-63464-z

**Published:** 2020-04-14

**Authors:** Kangjun Zhang, Vincent C. H. Lui, Yan Chen, Chun Nam Lok, Kenneth K. Y. Wong

**Affiliations:** 1 Department of Surgery, LKS Faculty of Medicine, University of Hong Kong, Hong Kong, China; 2 Department of Surgery, Guangzhou Women and Children’s Medical Centre, Guangzhou, China; 3 Department of Chemistry, Faculty of Science, University of Hong Kong, Hong Kong, China; 4 Present Address: The Third People’s Hospital of Shenzhen (The Second Affiliated Hospital of Southern University of Science and Technology), Shenzhen, China

**Keywords:** Experimental models of disease, Translational research

## Abstract

Burn injury is common, and antimicrobial agents are often applied immediately to prevent wound infection and excessive inflammatory response. Although inflammation is essential for clearing bacteria and creating an environment conducive to the healing process, it is unclear what time-frame inflammation should be present for optimal wound healing. This study critically investigated the role of early inflammation in burn wound healing, and also revealed the molecular mechanisms underlying the pro-healing effects of silver nanoparticles (AgNPs). We created a burn injury mouse model using wild-type and *Smad3*−/− mice, which were topically treated with AgNPs at different post-burn days, and examined the healing processes of the various groups. We also delineated the molecular pathways underlying the anti-inflammation and pro-healing effects of AgNPs by morphological and histological analysis, immuno-histochemistry, and western blotting. Our results showed that (1) AgNPs regulated pro-inflammatory cytokine IL-6 production of keratinocytes and neutrophils infiltration through KGF-2/p38 signaling pathway, (2) Topical AgNPs treatment immediately after burn injury significantly supressed early inflammation but resulted in delayed healing, (3) A short delay in AgNPs application (post-burn day 3 in our model) allowed early inflammation in a controlled manner, and led to optimal burn wound healing. Thus, our current study showed that some degree of early inflammation was beneficial, but prolonged inflammation was detrimental for burn wound healing. Further evaluation and clinical translation of this finding is warranted.

## Introduction

Wound repair is a cascade of events which involves initial clot formation by thrombocytes, migration of inflammatory cells, proliferation of epithelial cells and wound contraction^1,2^. The early stage of inflammation is a critical period in the wound healing process, and is essential for clearing bacteria and creating an environment conducive to the healing^3^. However, exaggerated inflammation can lead to destruction to the tissue and organs, systemic inflammatory response syndrome (SIRS), multiple organ dysfunction syndrome (MODS) or even death.

Early inflammatory response is the host defence that delivers a rapid reaction to an offending agent at the injured site. It leads to four major changes, which include (1) Vasodilatation to increase the local blood flow; (2) Structural changes of the microvasculature to allow the leukocytes and plasma proteins to leave the circulation; (3) Migration of leukocytes from the microcirculation to the injured site, and (4) Activation of leukocytes to remove the offending agents. Neutrophils are the major leukocytes being recruited from the microcirculation to the injured site after wounding at the early inflammation stage^4,5^. They migrate to the wound site within a few minutes after injury and accumulate there for a few days. The number of infiltrated neutrophils accumulated at the wound site is proportional to the severity and the extent of the injury. Neutrophils promote the removal of bacteria and necrotic tissue. Meanwhile, they also secrete pro-inflammatory cytokines such as interleukins 1 alpha and beta (IL-1α and 1β) and tumour necrosis factor alpha (TNF-α), which then activate a series of immune response, and stimulate epidermal cells to proliferate^6^.

Keratinocytes at the skin wound also secrete pro-inflammatory mediators including IL-1α, IL-6, IL-8, IL-15, IL-20, TNF-α, CXCL10 (IP-10), CCL5 (RANTES), CCL2 (MCP-1), and CCL20 (MIP-3α), and play an important role in the recruitment and activation of neutrophils^7–12^. IL-6 is an important cytokine involved in the early inflammation of burn injury, with its level peaking on post-burn day 3–4^13^. In skin burn wound, IL-6 is produced primarily by keratinocytes^14,15^. Using a burn injury model in rats, it was found that IL-6 level was the highest in full-thickness wound when compared to partial-thickness wound and sham control at 1 hour after burn injury, and this group also had much more neutrophils infiltration on post-burn day 7. This suggested that early elevation of IL-6 prolonged inflammation in full-thickness burn wound^16^. IL-6 further enhances the effect of TNF and IL-1, which combine to amplify the inflammatory response in the post-burn period^17–19^.

Mammalian p38 mitogen-activated protein kinases (MAPKs) consist of four members: p38α, p38β, p38γ and p38δ, and these MAPKs are activated by a wide range of cellular stresses as well as in response to inflammatory cytokines. MAPKs are activated upon dual phosphorylation by the dual specificity MAPK kinases (MEKs), the activated MAPK will then phosphorylate their downstream targets and regulate the biosynthesis and secretion of inflammatory cytokines including IL-1, IL-6, IL-8 and TNF-α^20–22^. Neutrophils express p38α. The p38 MAPK specific inhibitor SB203580 blocks the expressions of surface receptor of chemokine CXCR1 and surface adhesion molecules CD66b and CD11b, and abolishes the chemotactic migration of neutrophils^23^. Furthermore, inhibition of p38 MAP kinase leads to a marked reduction of neutrophil infiltration^24,25^. Human keratinocytes express p38α, p38β, and p38δ isoforms^26,27^. p38α and p38β are involved in response to stress and pro-inflammatory cytokines, while p38δ has been implicated in keratinocyte differentiation^28^.

Silver nanoparticles (AgNPs) are pure silver of less than 100 nanometers in diameter, which can be incorporated into the dressing, and provide a sustained release of Ag0 clusters and Ag+^29^. Moreover, due to the small size of the silver nanoparticles, there is a relatively larger surface area, with much more effective biological actions, such as antimicrobial effect, than any other silver salts, compounds and solutions^30,31^. Silver nanoparticles have broad-spectrum antibacterial activities which kill more than 650 kinds of micro-organisms within a few minutes. Most importantly, unlike antibiotics, it is much less likely for bacteria to develop resistance to silver nanoparticles^32^. However, the mechanism underlying anti-microbial activity of AgNPs is still largely not understood. It has been suggested that AgNPs may block the respiratory chain, as well as damage the DNA and cell wall of bacteria, leading to the death of bacteria^30,33^. Apart from its anti-microbial activity, the anti-inflammatory activity of AgNPs has also been demonstrated in skin wound^34–37^, intestine and bladder^38,39^. AgNPs suppress the production of the early pro-inflammatory cytokines, such as IL-6, but promote the production of an anti-inflammatory cytokine IL-10 at the skin wound in the early phase after injury^34^, which would indicate that AgNPs suppress early inflammation.

Inflammation is a normal process of wound healing, and early inflammation is a critical period during the wound healing process. However, the roles of early inflammation in the healing of wounds have been controversial. Inflammation has been shown to delay wound healing in incision wounds^5^. In contrast, suppressing inflammation by silver nanoparticles promoted wound healing in burn injury mouse models^34,35^, in full-thickness incision mouse model^40^, and in contact dermatitis porcine model^37^. To further address the roles of early inflammation in burn wound healing, we used a burn injury model in wild-type and *Smad3*−/− mice (which are unable to mount an inflammatory response), and topically applied AgNPs on different post-burn days, and examined the wound healing processes. We also investigated the molecular mechanisms underlying the wound healing promotion effects of AgNPs. Our current study showed that early inflammation was critical, but prolonged inflammation was adverse for burn wound healing. Indeed, a slight delay application of AgNPs to control inflammation promoted burn wound healing in mice.

## Materials and methods

### Mice

C57BL/6 N male mice (8–12 weeks; 24–30 grams) were purchased from the Laboratory Animal Unit, The University of Hong Kong. *Smad3* knockout mice were kindly provided by Professor LAN Huiyao, (Department of Medicine and Therapeutics, The Chinese University of Hong Kong. HKSAR, China). All mice were supplied with food and water *ad libitum*, and kept under pathogen-free conditions with a 12 h light/dark cycle. The whole experimentation was approved and supervised by Committee on the Use of Live Animals in Teaching & Research, The University of Hong Kong (CULATR No.: 2341-11). All experimental mice were anesthetized by intra-peritoneal injection of pentobarbital sodium solution at a dose of 50 mg/kg (Abbott Laboratories, USA). All experiments were performed in accordance with relevant guidelines and regulations.

### Burn wound preparation and treatment

C57BL/6 N male mice were divided into four groups (5 mice in each group): AgNPs treatment started immediately after burn injury (at day 0); on day 3 or day 5 after burn injury, and no AgNPs treatment group. A burn wound (3 × 2 cm^2^) was created on the dorsal skin according to the protocol as described previously^41^.

In another set of experiments using *Smad3*−/− mice, these were divided into AgNPs treatment group and no treatment group. As *Smad3*−/− mice are significant smaller in body size when compared to their wild-type littermates, they could not survive a big burn wound (3 × 2 cm^2^). A smaller burn wound (1 × 1 cm^2^) was thus created on *Smad3*−/− mice experiments.

For all animals, the wounds were covered with surgical dressings and bandage. These were changed every three days. For the AgNPs group, AgNPs-containing suspension (1 mmol/L) was added to the surgical dressings and topically applied to the wound bed^42^. The wound areas were photographed with identical settings at the time of surgical dressing change. The wound areas were calculated using Photoshop CS software (Adobe, USA), and the healing rate at each time point of each group was expressed as the ratio of size of the wound area at different time point versus the size of the wound area at day 0 (Wound area = Wound area size on day n/Wound area size on day 0 × 100%). Healing of the wound was defined as the wound completely covered by fibrosis tissue^41^.

### Histology and Immumohistochemical staining

In experiments where wild type mice were used, the animals were sacrificed on 3, 7 and 10 days post injury. In *Smad3*−/− mice experiments, the animals were sacrificed on day 3 after wounding. Burn wound skin samples were collected for immunohistochemistry. Specimens were fixed in 4% paraformaldehyde/PBS overnight and embedded in paraffin for sectioning (4~6 µm in thickness). For histology staining, sections were stained with hematoxylin and eosin.

As for neutrophil, specimens were treated with rat anti-mouse primary antibody (Anti-Neutrophil Elastase, Abcam Ltd., USA, 1:100, 4 °C overnight), and rabbit anti-rat secondary antibody (Dako Ltd., USA, 1:200, 37 °C for 1 hour). As for IL-6 (interleukin-6), sections were treated with rat anti-mouse primary antibody (Invitrogen Ltd., USA, 1:100, 4 °C overnight), followed by rabbit anti-rat secondary antibody (Dako Ltd., USA, 1:200, 37 °C for 1 hour). DAB substrate kit (Vector Laboratories, USA) was used for color development following the manufacturer’s protocol. The sections were counterstained with hematoxylin, mounted with DPX mountant (BDH PROLABO^®^) and examined.

To quantify neutrophils infiltration, 5 continuous × 200 microscopic views at the wound margin were captured (Nikon, Tokyo, Japan). The immuno-reactive cells were counted in each view, and a mean value was calculated.

### Immuno-fluorescence staining

Tissues were harvested and processed as described above. For p-p38 staining, specimens were treated with rabbit anti-mouse primary antibody (Cell Signaling Technology, USA, 1:800, 4 °C overnight), and goat anti-rabbit IgG (H + L) secondary antibody, Alexa Fluor^®^ 488 conjugate (Thermo Fisher Scientific, USA, 1:200, 37 °C for 1 hour). For neutrophil staining, specimens were treated with rat anti-mouse primary antibody (Anti-Neutrophil Elastase, Abcam Ltd., USA, 1:100, 4 °C overnight), and goat anti-rat IgG (H + L) secondary antibody, Alexa Fluor^®^ 633 conjugate (Thermo Fisher Scientific, USA, 1:200, 37 °C for 1 hour). For KGF-2/FGF-10 staining, sections were treated with sheep anti-mouse primary antibody (R&D Systems, USA, 10 µg/mL, 4 °C overnight), followed by Alexa Fluor 633 conjugated donkey anti-sheep IgG secondary antibody (Thermo Fisher Scientific, USA, 1:200, 37 °C for 1 hour). Images were captured at the wound margin for evaluation (×200 magnification).

### Western blot analysis

Mouse skin tissues (at the junction of normal and scar) of each group were isolated for western blot analysis. Samples were homogenized and proteins were extracted using RIPA buffer according to manufacturer’s protocol (Cell Signaling Technology, USA, for 30 minutes). Proteins were separated by SDS-PAGE (10% gel) and transferred onto polyvinylidene difluoride (PVDF) membrane (Sigma-Aldrich, USA). The membrane was blocked in 5% bovine serum albumin (BSA) for 2 hours at room temperature. Then, the blots were incubated with p-p38 (Cell Signaling Technology, USA, 1:1000, 4 °C overnight) or KGF-2 antibodies (R&D Systems, USA, 1 µg/mL, 4 °C overnight) in 5% BSA solution, followed by incubation with secondary antibodies conjugated with horseradish peroxidase for 1 hour. Signals were visualized with enhanced chemiluminescence (ECL) and exposure to Hyperfilm (Amersham Biosciences, UK). *β*-actin expression was served as a loading control.

### Statistical analyses

Data was indicated as mean ± SEM (standard error of mean). Statistical comparisons were performed by using ANOVA by means of Bonferroni test for comparisons between groups; *p* < 0.05 were considered as a significant difference.

## Results

### Burn wound healing is accelerated by delayed application of AgNPs

**Figure 1:**
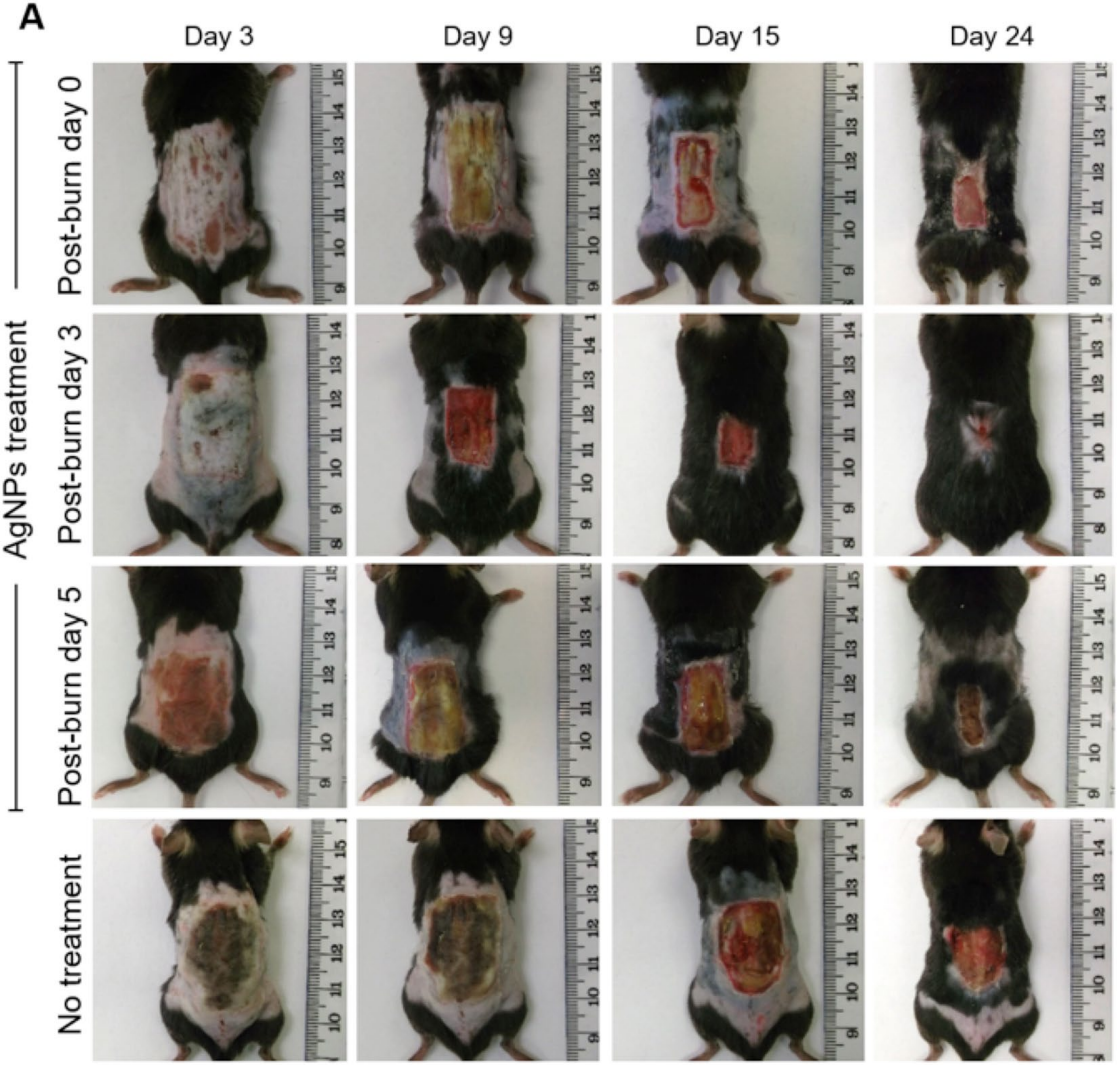
Delayed AgNPs treatment at post-burn day 3 led to a faster wound healing. (**A**) Photos of the burn wounds of different AgNPs treatment groups (started at post-burn day 0, 3 and 5) and untreated (no treatment) group at post-burn day 3, 9, 15 and 24.

To investigate if the commencement of AgNps treatment at different post-burn days affect burn wound healing, we compared the rate of wound healing in mice receiving AgNPs at post-burn day 0, 3, or 5 and wounds receiving no AgNPs (untreated). As shown in Fig. 1A, when compared with the untreated group when healing took around 40.4 ± 0.8 days, the duration of wound healing was significantly shortened when AgNPs was applied to the wound at day 0 (35.8 ± 0.6 days) (*p* < 0.05) (Table 1). This data again corroborated our previous report that AgNPs accelerated burn wound healing^28^. However, the burn wound healed in even a shorter period if AgNPs treatment was started at day 3 (27.8 ± 0.7 days), which was significantly shorter than each of the other three groups (post-burn day 0 and 5 treatment groups; untreated group) (*p* < 0.05) (Table 1). It is noteworthy mentioning that the healing of post-burn day 5 treatment burn wounds (healed in 35.8 ± 0.8 days) was significantly longer than that of post-burn day 3 treatment wounds (*p* < 0.05) (Table 1). Taken all together, these indicated that a delayed application of AgNPs by 3 days after burn injury resulted in the fastest healing.

**Table 1:** Comparison of wound healing times in different treatment groups.

Days of Would Healing (Mean ± S.E.M.)			
No Treatment	Post-burn Day 0	Post-burn Day 3	Post-burn Day 5
40.4 ± 0.8	35.8 ± 0.6 (*p*: 0.02^a*^)	27.8 ± 0.7 (*p*: 0.000^a*^; 0.000^b*^)	35.8 ± 0.8 (*p*: 0.02^*a^; 1.000^b^; 0.000^*c^)

Note: ^a^*p* value of comparison with no treatment group; ^b^*p* value of comparison with post-burn day 0 group; ^c^*p* value of comparison with post-burn day 3 treatment group. *(*p* < 0.05; statistical significance).

To better quantify the efficacy of AgNPs treatment strategies in the promotion of burn wound healing, we determined the percentages of wound healed in different groups. As shown in Table 2, on day 21, the percentage of wound area was 27.0 ± 1.6% and 28.8 ± 0.9%, respectively in the wounds treated with AgNPs immediately and 5 days after injury, whereas the wound area was only 5.4 ± 0.2% in mice treated with AgNPs 3 days after injury (*p* < 0.01). Meanwhile, the wounds of untreated mice only reduced to 46.9 ± 2.5% at the same time point, which was significantly larger than all the AgNps treatment groups (*p* < 0.05). This further confirmed that a delayed application of AgNPs (post-burn day 3) was the most effective in promoting burn wound healing amongst all the treatment groups.

**Table 2:** Comparison of rates of wound healing in different treatment groups.

	% of Original Wound Area (Mean ± S.E.M.)			
	No Treatment	Post-burn Day 0	Post-burn Day 3	Post-burn Day 5
Day 6	96.5 ± 1.2	97.1 ± 0.7 (*p*: 1.000^a^)	86.7 ± 2.7 (*p*: 0.077^a^; 0.055^b^)	87.2 ± 3.9 (*p*: 0.098^a^; 0.071^b^; 1.000^c^)
Day 9	91.3 ± 2.3	77.0 ± 0.7 (*p*: 0.012^a*^)	60.5 ± 4.1 (*p*: 0.000^a*^; 0.004^b*^)	81.4 ± 2.7 (*p*: 0.130^a^; 1.000^b^; 0.000^c*^)
Day 15	65.6 ± 1.2	43.0 ± 2.8 (*p*: 0.000^a*^)	26.9 ± 1.4 (*p*: 0.000^a*^; 0.000^b*^)	53.7 ± 1.4 (*p*: 0.002^a*^; 0.004^b*^; 0.000^c*^)
Day 21	46.9 ± 2.5	27.0 ± 1.6 (*p*: 0.000^a*^)	5.4 ± 0.2 (*p*: 0.000^a*^; 0.000^b*^)	28.8 ± 0.9 (*p*: 0.000^a*^;1.000^b^; 0.000^c*^)
Day 27	26.8 ± 4.0	17.8 ± 0.8 (*p*: 0.046^a*^)	0.4 ± 0.2 (*p*: 0.000^a*^; 0.029^b*^)	10.1 ± 0.8 (*p*: 0.000^a*^; 0.110^b^; 0.029^c*^)

Note: ^a^*p* value of comparison with no treatment group; ^b^*p* value of comparison with post-burn day 0 group; ^c^*p* value of comparison with post-burn day 3 treatment group. ^*^(*p* < 0.05; statistical significance).

**Figure 2:**
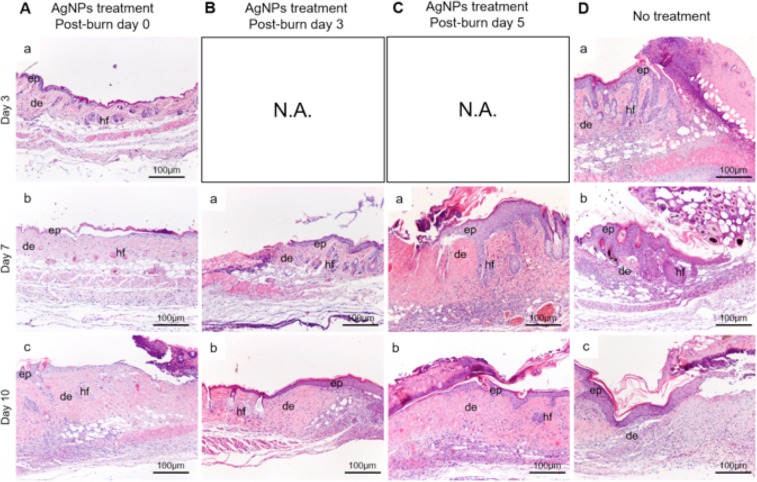
Histological examination of burn wounds of AgNPs treatment groups started at post-burn day 0 (**A**), post-burn day 3 (**B**) and post-burn day 5 (**C**), and no treatment (**D**) at post-burn day 3, 9, 15 and 24. Abbreviations: ep, epidermis; de, dermis; hf, hair follicle. N.A.: Not Applicable.

To examine and compare the wound healing processes of each group, we performed H&E staining of the sections from the edge of the wounds of different groups. In the group that received AgNPs immediately after burn (day 0), the post-burn day 3, 7 and 10 wounds displayed thinner epidermis, fewer infiltrated cells and fewer necrotic tissues (Fig. 2A) when compared with that of the wounds treated with AgNPs starting on post-burn day 3, 5 (Fig. 2B,C) and untreated wounds (Fig. 2D). On post-burn day 7, thickened epidermis could be seen to have migrated under the necrotic tissues in the wounds of animals treated with AgNPs starting on post-burn day 5 (Fig. 2C–a), and in the untreated wounds (Fig. 2D–b). In addition, infiltrated cells were abundant in the thickened dermis as compared with the wounds treated with AgNPs on post-burn day 0 (Fig. 2A-b) and post-burn day 3 (Fig. 2B-a). On post-burn day 10, the epidermis became less thick in the wounds treated with AgNPs starting at post-burn day 5 (Fig. 2C-b) and in the untreated wounds (Fig. 2D-c), yet infiltrated cells were still more than that in the wounds treated with AgNPs at post-burn day 0 (Fig. 2A-c) and at post-burn day 3 (Fig. 2B-b).

### Regulation of inflammation at the early post-injury phase results in faster burn wound healing

**Figure 3:**
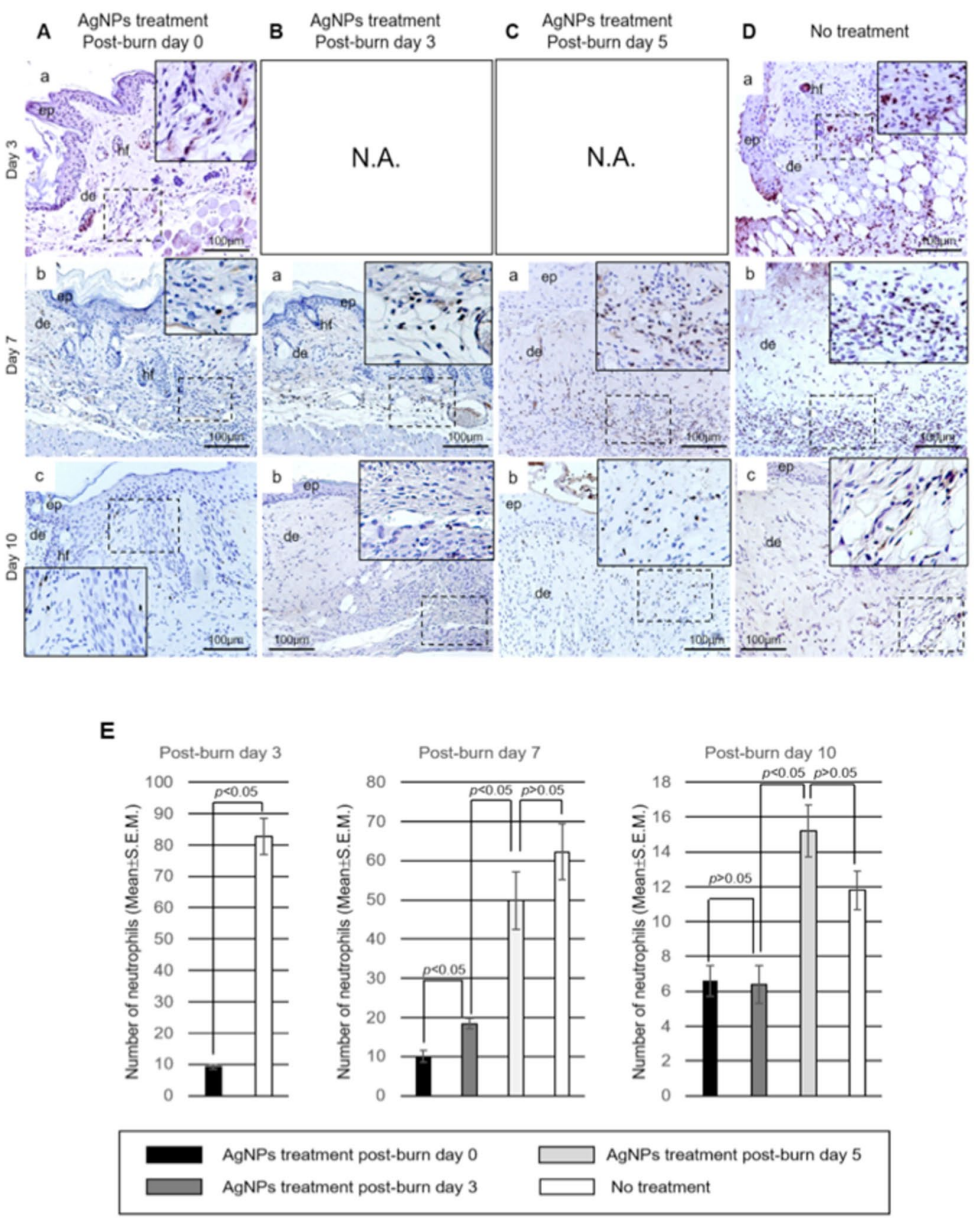
Reduced neutrophil infiltration of burn wounds by AgNPs. Sections of wounds of AgNPs treatment groups started at post-burn day 0 (**A**), post-burn day 3 (**B**) and post-burn day 5 (**C**), and no treatment (**D**) at post-burn day 3, 7 and 10 were stained for neutrophil elastase (brown). Regions highlighted with dotted line were magnified and shown as inset. (**E**) Average number of neutrophils per a 200X microscopic view at the wound margin of wounds of different AgNPs treatment groups (started at post-burn day 0, 3 and 5) at post-burn day 3, 7 and 10 were quantified and shown as mean ± S.E.M. Abbreviations: ep, epidermis; de, dermis; hf, hair follicle. N.A.: Not Applicable.

To investigate if the anti-inflammatory action of AgNPs attributed to the promotion of burn wound healing, we examined neutrophils infiltration and inflammatory cytokine interleukin-6 (IL-6) expression. On post-burn day 3, there were significantly fewer neutrophils infiltrated into the wounds in the AgNPs post-burn Day 0 treatment group (Fig. 3A-a) when compared with to untreated wounds (*p* < 0.05) (Fig. 3D-a). On post-burn day 7, wounds treated with AgNPs on post-burn day 0 and 3 (Fig. 3A-b,B-a) showed fewer infiltrated neutrophils than the other two groups (Fig. 3C-a,D-b). In addition, the number of infiltrated neutrophils increased proportionally in relation to when the AgNPs treatment was started post-burn (*p* < 0.05) (Fig. 3E, Supplementary Table 1). On post-burn day 10, the number of infiltrated neutrophils decreased markedly as compared to their respective groups on post-burn day 7. The numbers of infiltrated neutrophils were similar between the groups with AgNPs treatment starting at post-burn day 0 and 3, and the numbers of infiltrated neutrophils were similar between the group treated with AgNPs starting at post-burn day 5 and the untreated group. However, the groups treated with AgNPs at post-burn day 0 and 3 still had significantly lower number of infiltrated neutrophils than that of the group treated with AgNPs starting at post-burn day 5 and the untreated group (Fig. 3E and Supplementary Table 1).

**Figure 4:**
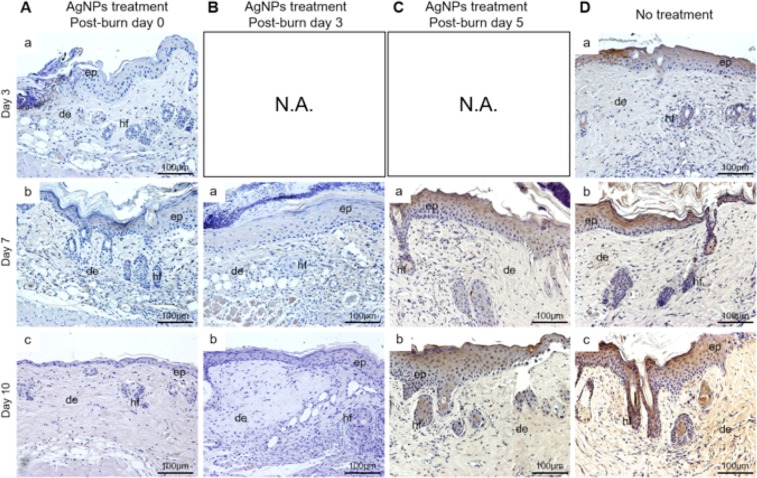
Reduced IL-6 expression of burn wounds by AgNPs. Sections of wounds of AgNPs treatment group started at post-burn day 0 (**A**), post-burn day 3 (**B**) and post-burn day 5 (**C**), and no treatment (**D**) group at post-burn day 3, 7 and 10 were stained for IL-6 (brown). Abbreviations: ep, epidermis; de, dermis; hf, hair follicle. N.A.: Not Applicable.

The expression of IL-6 was markedly reduced in the wounds treated with AgNPs starting at post-burn day 0 and day 3. AgNPs failed to reduce IL-6 expression if it was applied at post-burn day 5. The expression of IL-6 was mainly localized to the epidermis (Fig. 4). Since the keratinocytes constituted about 90% of the cells of the epidermis^15^, which indicated that IL-6 was produced primarily by keratinocytes in the epidermis of the wounds in our study. Taken all these together, it would suggest that the fastest wound healing seen when AgNPs were applied on post-burn day 3 could be due to the attenuation of inflammatory responses at the early stage of inflammation.

### AgNPs regulated neutrophil infiltration via modulation of KGF-2/p38 signaling

**Figure 5:**
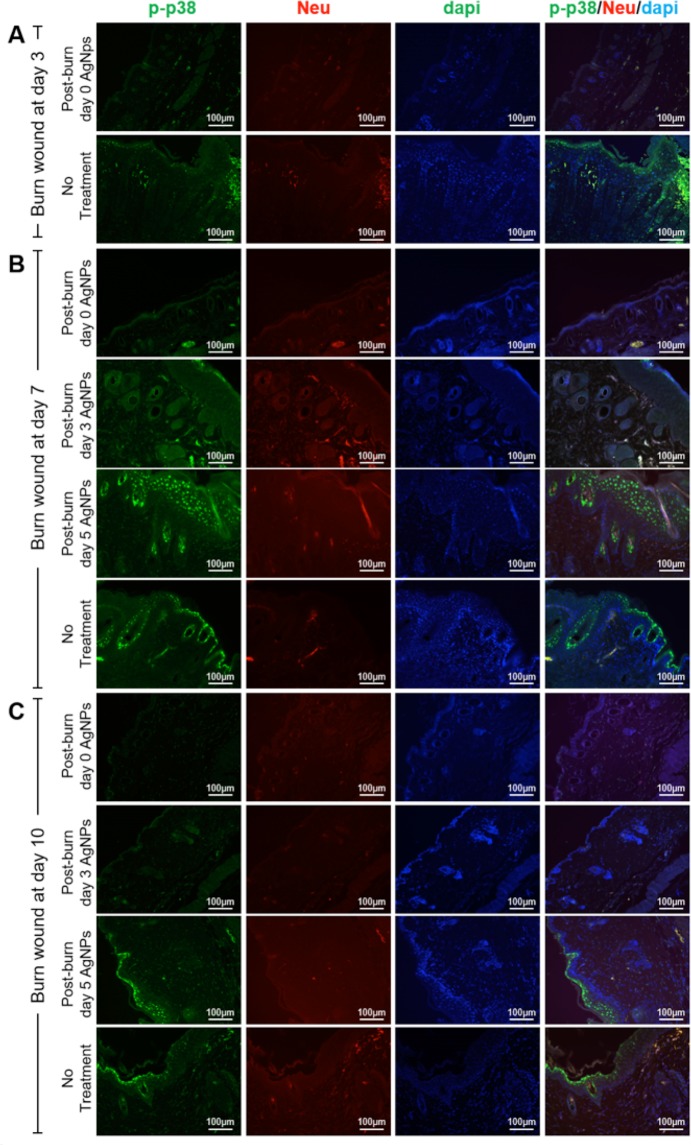
Reduced p-38 phosphorylation of burn wounds by AgNPs. Sections of post-burn day 3 (**A**), post-burn day 7 (**B**) and post-burn day 10 (**C**) wounds of AgNPs treatment group (started at post-burn day 0, 3 and 5) and no treatment group were co-stained for p-p38 (green), neutrophil elastase (Neu; red) and counter-stained with dapi for nuclei.

To study if AgNPs reduced neutrophil infiltration by the modulation of the phosphorylation of p38, we examined the expression of phosphorylated p38 (p-p38) in the wounds. Co-staining for p-p38 and neutrophils revealed that p-p38 expression was mainly localized to the keratinocytes in the epidermis and in the hair follicles, and also to the neutrophils (Fig. 5). The immuno-fluorescence of p-p38 was the highest in the untreated wound on post-burn day 7 (Fig. 5B), and the expression declined on post-burn day 10 (Fig. 5C). The immuno-fluorescence of p-p38 at the epidermis and at the hair follicles was largely reduced in the wounds treated with AgNPs on post-burn day 0 and 3. However, the wounds treated with AgNPs at post-burn day 5 showed comparable the immuno-fluorescence of p-p38 with the untreated wounds (Fig. 5B,C). This indicated that AgNPs given after day 5 of burn injury failed to suppress p38 phosphorylation.

**Figure 6:**
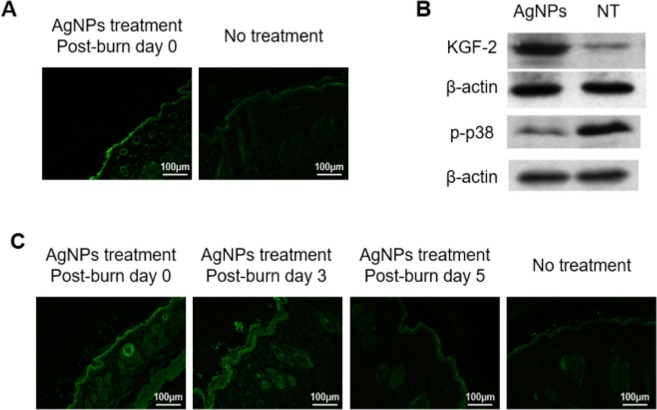
Elevated KGF-2 expression of burn wounds by AgNPs. Sections of post-burn day 3 (**A**) and post-burn day 7 (**C**) wounds of AgNPs treatment group (started at post-burn day 0, 3 and 5) and no treatment group were stained for KGF-2 (green). (**B**) Western blot analysis of the post-burn day 3 wound of AgNPs treatment started at post-burn day 0 and no treatment (NT) for KGF-2 and p-p38 expressions. Expression level of β-actin was used as loading controls for samples.

To further delineate the molecular mechanisms underlying the suppression of p38 phosphorylation, we tested the KGF-2, an upstream regulator of p38^42^. Immuno-fluorescence of KGF-2 was mainly localized at the epidermis and at the hair follicles. AgNPs treatment at post-burn day 0 up-regulated the KGF-2 expression of the post-burn day 3 wound as revealed by both immuno-fluorescence staining and western blot analysis (Fig. 6A,B). At the post-burn day 7, up-regulation of KGF-2 was observed in the groups treated with AgNPs at post-burn day 0 and 3. However, the wounds treated with AgNPs at post-burn day 5 showed comparable the immuno-fluorescence of KGF-2 with the untreated wounds (Fig. 6C). Combine with the data of p-p38 above, the increasing pattern of KGF-2 is consistent with the decreasing mode of p-p38 after burn injury, which highly indicating the KGF-2/p38 signaling pathway is involved in the AgNPs regulated neutrophil infiltration.

### AgNPs failed to promote wound healing in *Smad3*−/− mice

**Figure 7:**
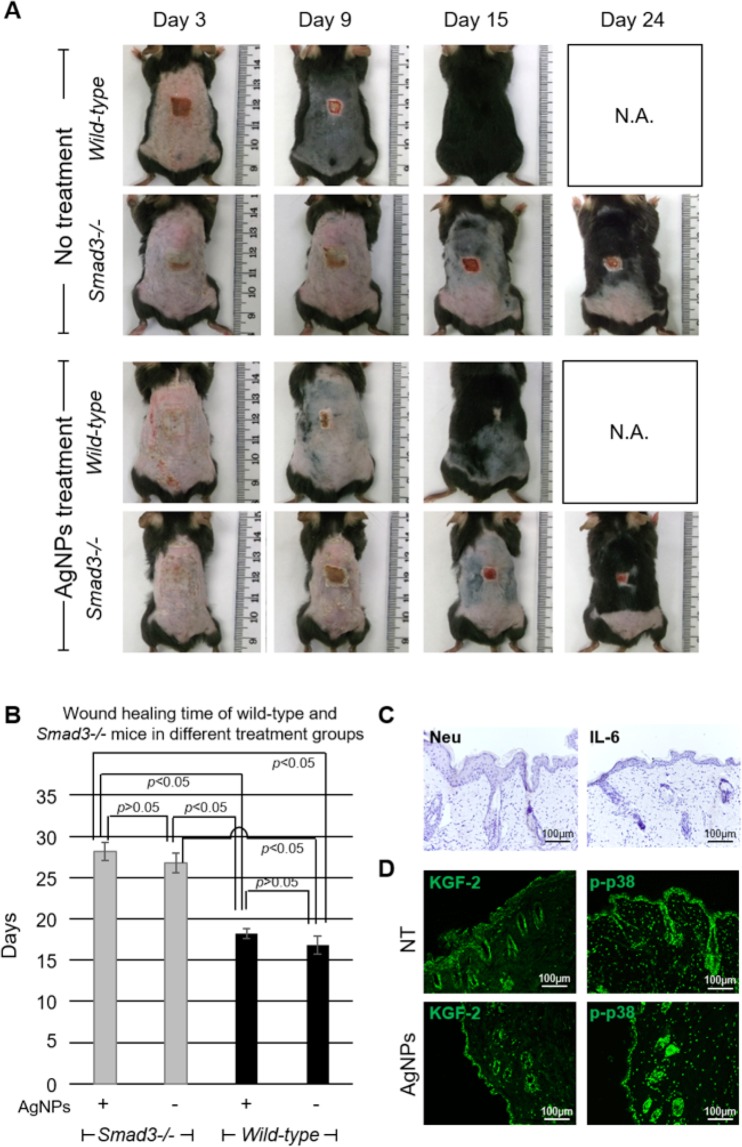
Burn wound healing is delayed in *Smad3*−/− mice. (**A**) Photos of the burn wounds of wild-type and *Smad3*−/− mice with AgNPs treatment (started at post-burn day 0) or without AgNPs treatment (No treatment) at post-burn day 3, 9, 15 and 24. (**B**) Wound healing times of wild-type and *Smad3*−/− mice with or without AgNPs treatment were shown for comparison. (**C**) Sections of post-burn day 3 wounds of *Smad3*−/− mice were stained for neutrophil elastase (Neu; brown) and IL-6 (brown). (D) Sections of post-burn day 3 wounds of *Smad3*−/− mice with (AgNPs) or without AgNPs treatment (NT) were stained for KGF2 (green) and p-p38 (green). N.A.: Not Applicable.

To further confirm the need of inflammation in burn wound healing, we performed the same burn wound experiment using *Smad3* knockout (*Smad3*−/−) mice, which were unable to mount an inflammatory response. The wound healing time was comparable between *Smad3*−/− mice with and without AgNPs treatment (28.2 ± 1.1 days versus 26.8 ± 1.2 days) (Supplementary Table 2). Furthermore, the wound healing time was significantly longer in *Smad3*−/− mice as compared with the wild-type mice (*p* < *0.05*) (Fig. 7A,B). As shown in Fig. 7C, neutrophils and IL-6 expression were largely undetected in the post-burn day 3 wounds of the *Smad3*−/− mice. This result indicated that inflammation was important in burn wound healing and lack of early inflammatory response led to delayed burn wound healing.

Moreover, as shown in Fig. 7D, the increasing of KGF2 by AgNPs failed to suppress the expression of p-p38 in burn wounds of *Smad3*−/− mice, indicating the Smad3 may play a key role in KGF2 regulated p-p38, and loss of Smad3 block the inhibition effect of KGF2 on p-p38. In addition, the high expression of p-p38 in burn wounds failed to induce the local inflammatory response in *Smad3*−/− mice (Fig. 7C,D), which suggested the Smad3 also participated in the p-p38 initiated pro-inflammatory cascade reaction after burn injury in mice.

## Discussion

The early stage of inflammation is a critical period of the wound healing process, essential for clearing contaminating bacteria and creating an environment conducive to facilitate the healing process^3^. In the absence of effective decontamination, however, inflammation may be prolonged, the wound may enter into a chronic state and fail to heal. This is particularly true for burn wounds. Our current study demonstrated that early inflammation was indeed beneficial, but prolonged inflammation was detrimental for healing, and a slight delay in application of silver nanoparticles (AgNPs) further promoted burn wound healing in mice.

Silver nanoparticles (AgNPs) suppressed the inflammatory cytokine IL-6 production of keratinocytes and neutrophil infiltration of the burn wounds, and promoted would healing in mice. Comparison among the wound healing rates of AgNPs treatment starting on post-burn days 0, 3 and 5, revealed that a short delay in AgNPs treatment (commencement at the post-burn day 3) resulted in the fastest healing. AgNPs treatment starting at the post-burn day 0 and 3 drastically suppressed IL-6 production and neutrophils infiltration, while AgNPs treatment starting at the post-burn day 5 failed to do so. Therefore, the effective window of AgNPs in the promotion of burn wound healing and suppression of local inflammatory responses could be somewhere after post-burn day 1 but before day 5 in the current model. Productions of IL-6 by keratinocytes and neutrophil infiltration were markedly reduced in the *Smad3*−/− wounds, and AgNPs failed to promote burn wound healing of *Smad3*−/− mice. Furthermore, burn wound healing in *Smad3*−/− mice was significantly delayed as compared to the wild-type mice. *Smad3*−/− mice displayed diminished mucosal immunity and impaired T cell response, and died within a few months of severe immunodeficiency and infection^44^. In line with the diminished immunity of *Smad3*−/− mice, the *Smad3*−/− burn wound failed to elicit local inflammatory responses including inflammatory cytokine IL-6 production and neutrophils infiltration. Taken all these together, it would indicate that (1) an early and local inflammation is beneficial in the promotion of healing of burn wound, and (2) a prolonged local inflammation is detrimental to burn wound healing.

Mammalian p38 mitogen-activated protein kinases (p38 MAPKs) regulates the production of inflammatory mediators, and plays an important role in inflammation. Once activated by cellular stresses and in response to inflammatory cytokines, p38 MAPKs are activated upon phosphorylation by MMKs^43^. KGF-2 suppresses MMKs and p38 MAPK signalling via the regulation of ROS and ASK1^45,46^. AgNPs markedly induced KGF-2 expression, and in turn reduced the phosphorylation of p38 of the epidermis of the burn wounds. AgNPs has also been shown to up-regulate KGF-2 in a contact dermatitis porcine model^34^. Hence this would indicate that AgNPs can stimulate the expression of KGF-2 before three days in the inflammatory skin after wounding. By suppressing the phosphorylation activation of p38 MAPK, AgNPs led to a reduction of IL-6 production of the keratinocytes, and subsequently less neutrophil infiltration in the burn wounds. Furthermore, KGF-2 has also been shown to stimulate the proliferation and migration of keratinocytes, and promote detoxification of reactive oxygen species (ROS), which protects keratinocytes from ROS-induced apoptosis^45,46^. Thus, pro-healing activity could also have been partly attributed to the enhanced re-epithelialization rates as a result of up-regulation of KGF-2 by AgNPs in the current and our previous studies^34,35,40^.

In conclusion, current findings indicate that (1) AgNPs regulate the inflammatory response through KGF2/p38 signaling pathway, (2) Topical treatment of AgNPs immediately after burn injury supresses the early inflammation, and (3) A short delay of AgNPs treatment (post-burn day 3 in our current model) allows early inflammation but prevents the prolonged inflammation, which results in optimal burn wound healing. In the clinical management of burn wounds, clinicians have always inclined to apply anti-bacterial and anti-inflammation treatment immediately after wounding with the aim to prevent excessive inflammatory response. Our findings here seem to challenge this dogma. Thus, a clinical trial to compare the efficacy of immediate versus delayed application of silver nanoparticles or other anti-inflammation drugs in the promotion of burn wound healing is eagerly awaited.

## Supplementary information

Supplementary Tables

## Data Availability

The data generated during and/or analyzed during the current study are available from the authors upon reasonable request.
