# Supplementary material for: Delayed application of silver nanoparticles reveals the role of early inflammation in burn wound healing
Source: Sci Rep. 2020 Apr 14;10:6338. doi: 10.1038/s41598-020-63464-z (PMC7156632; doi:10.1038/s41598-020-63464-z)
Supplement: Supplementary file 1 — Supplementary Tables [file 41598_2020_63464_MOESM1_ESM.pdf]

Delayed application of silver nanoparticles reveals the role of early inflammation in burn wound healing

Kangjun Zhang, MD, Ph.D[a#], Vincent C.H. Lui, PhD[a], Yan Chen, PhD[b], Chun Nam Lok, PhD[c], Kenneth K.Y. Wong, MD, PhD[a,*]

Supplementary Table 1. Neutrophils infiltration in different treatment groups.

	Neutrophils Number (Mean±S.E.M.)			
	**No Treatment**	**Post-burn Day 0**	**Post-burn Day 3**	**Post-burn Day 5**
**Day 3**	82.8±5.8	9.2±0.7 (*p*: 0.000[a]*)	N.A.	N.A.
**Day 7**	62.2±7.1	10.0±1.5 (*p*: 0.000[a]*)	18.4±1.3 (*p*: 0.000[a]*; 1.000[b])	49.8±7.4 (*p*: 0.672[a]; 0.000[b]*; 0.004[c]*)
**Day 10**	11.8±1.1	6.6±0.9 (*p*: 0.038[a]*)	6.4±1.1 (*p*: 0.030[a]*; 1.000[b]*)	15.2±1.5 (*p*: 0.343[a]; 0.001[b]*; 0.000[c]*)

Note: [a]: *p* value of comparison with no treatment group; [b]: *p* value of comparison with post-burn day 0 group; [c]: *p* value of comparison with post-burn day 3 treatment group. *: ($p<0.05$; statistical significance). N.A.: Not Applicable.

Supplementary Table 2. Comparison of wound healing times of *wild-type* and *Smad3-/-* mice in different treatment groups.

	**Days of Would Healing (Mean±S.E.M.)**	
	**No Treatment**	**AgNPs**
Wild-type	16.8±1.1	18.2±0.6 (*p*: 1.000[a])
Smad3-/-	26.8±1.2 (*p*: 0.000[a]*; 0.000[b]*)	28.2±1.1 (*p*: 0.000[a]*; 0.000[b]*; 1.000[c])

Note: [a]: *p* value of comparison with *wild-type* no treatment group; [b]: *p* value of comparison with *wild-type* AgNPs treatment group; [c]: *p* value of comparison with *Smad3-/-* no treatment group. *: ($p<0.05$; statistical significance).
